# Dysfunction of the Autonomic Nervous System and its Role in the Pathogenesis of Septic Critical Illness (Review)

**DOI:** 10.17691/stm2020.12.4.12

**Published:** 2020-08-27

**Authors:** Y.Y. Kiryachkov, S.A. Bosenko, B.G. Muslimov, M.V. Petrova

**Affiliations:** Head of the Department of Surgical and Resuscitation Technologies; Federal Research and Clinical Center of Intensive Care Medicine and Rehabilitology, 25, Bldg 2, Petrovka St., Moscow, 107031, Russia;; Anesthesiologist; Federal Research and Clinical Center of Intensive Care Medicine and Rehabilitology, 25, Bldg 2, Petrovka St., Moscow, 107031, Russia;; Deputy Chief Physician for Anesthesiology and Intensive Care; Konchalovsky Central City Hospital, 2, Bldg 1, Kashtanovaya Alley, Zelenograd, Moscow, 124489, Russia; Professor, Deputy Director Federal Research and Clinical Center of Intensive Care Medicine and Rehabilitology, 25, Bldg 2, Petrovka St., Moscow, 107031, Russia;

**Keywords:** autonomic nervous system, sepsis, cholinergic anti-inflammatory pathway.

## Abstract

Dysfunction of the autonomic nervous system (ANS) of the brain in sepsis can cause severe systemic inflammation and even death. Numerous data confirmed the role of ANS dysfunction in the occurrence, course, and outcome of systemic sepsis. The parasympathetic part of the ANS modifies the inflammation through cholinergic receptors of internal organs, macrophages, and lymphocytes (the cholinergic anti-inflammatory pathway). The sympathetic part of ANS controls the activity of macrophages and lymphocytes by influencing β2-adrenergic receptors, causing the activation of intracellular genes encoding the synthesis of cytokines (anti-inflammatory beta2-adrenergic receptor interleukin-10 pathway, β2AR–IL-10). The interaction of ANS with infectious agents and the immune system ensures the maintenance of homeostasis or the appearance of a critical generalized infection. During inflammation, the ANS participates in the inflammatory response by releasing sympathetic or parasympathetic neurotransmitters and neuropeptides. It is extremely important to determine the functional state of the ANS in critical conditions, since both cholinergic and sympathomimetic agents can act as either anti- or pro-inflammatory stimuli.

## Introduction

The functional state of the autonomic nervous system (ANS) plays an important role in the regulation of the inflammatory response in the body [1]. With this in mind, the pathogenicity of the causative agent, its appearance, and the route of invasion cannot be considered the main factors in the occurrence and development of a generalized infection. It is the neuroendocrine and immune responses of the body to bacterial invasion that have a decisive influence on the development and prognosis of the septic state, multiple organ dysfunction, and mortality.

## Pathophysiological role of the ANS in the development of sepsis and septic shock

### ANS dysfunction is the pathophysiological trigger of sepsis.

In septic conditions, systemic inflammatory response syndrome (SIRS) and multiple organ dysfunction syndrome (MODS) manifest most heavily when the ANS is out of control [2]. The ANS dysfunction serves as the main trigger for pathophysiological and clinical manifestations of sepsis [3–5]. The functionality of the ANS is based on two regulatory components: vagal (parasympathetic) and sympathoadrenal (sympathetic) mechanisms. Multiple data indicate that enhancement of the parasympathetic component reduces inflammatory manifestations through the implementation of the so-called cholinergic anti-inflammatory pathway (CAP). A number of publications described the CAP-dependent systemic and local inflammatory reactions [6, 7]. With the development of inflammation, an afferent impulse is transmitted along the vagal nerve from the periphery to the brain stem. In the opposite direction, the vagal efferent pathway transmits signals to the spleen, liver, intestines, and other organs [8–16]. Stimulation of the efferent vagal nerve, as well as the use of cholinesterase blockers, results in accumulation of acetylcholine in these organs. In turn, acetylcholine interacts with the nicotinic acetylcholine receptors (nAChR), in particular with the nicotinic acetylcholine receptor α7 (α7nAChR), which is expressed by macrophages and other cytokineproducing cells. Acetylcholine reduces the production of TNFα by human macrophages in a dose-dependent manner, and this anti-inflammatory effect is mediated by the α7 subunit of the nicotinic cholinergic receptor. Acetylcholine stimulation of α7nAChR has an inhibitory effect on white blood cells, including macrophages and type 2 congenital lymphoid cells. The latter are a group of lymphocytes that are involved in the rapid cytokine-dependent response in the body during the inflammatory process; unlike regular acquired immunity lymphocytes, they lack antigen-specific receptors and can respond to a wide range of inflammation stimuli. Ultimately, the concentration of pro-inflammatory cytokines is markedly inhibited. This neuroimmune message is the cholinergic anti-inflammatory pathway [17].

It is important to note that the sympathetic arm of the ANS is also actively involved in the control of inflammatory reactions. The mechanism of neurogenic inhibition of inflammation is reportedly based on the production of norepinephrine by catecholaminergic nerve fibers in the spleen. Norepinephrine binds to β2-adrenergic receptors (β2AR) of CD4^+^ T cells (T-helpers). CD4^+^ triggers the release of acetylcholine, which inhibits the secretion of inflammatory cytokines in macrophages by activating the α7nAChR followed by the implementation of the CAP. In addition, β2AR agonists can increase the production of IL-10 by myeloid cells that have anti-inflammatory properties; the mechanism is known as the β2AR–IL-10 [18]. The role of β2-agonists (mediators of the sympathetic nervous system) in blocking the systemic inflammatory reactions, which in turn, leads to the inhibition of macroangiopathies, has been exemplified by the phenomenon of macrophage activation in persistent diabetes [19]. β2AR agonists act as potent inhibitors of TNF-α production by bone marrow macrophages. The anti-inflammatory effect of β2AR manifests, for example, in a model of acute cerebrovascular accident. Ischemic stroke provokes a neuroinflammatory process and the prolonged release of epinephrine and norepinephrine by the sympathetic nervous system. The enhanced β2-adrenergic signaling after the onset of stroke is known to suppress the involvement of microglia, reducing the activation of both pro-inflammatory and anti-inflammatory cytokines. In contrast, a decrease in β2-adrenergic signaling in the microglia increases both pro-inflammatory and anti-inflammatory cytokine expression after stroke. Therefore, β2AR can be a therapeutic target for reducing the inflammation and improving the post-stroke recovery [20].

**Figure 1:**
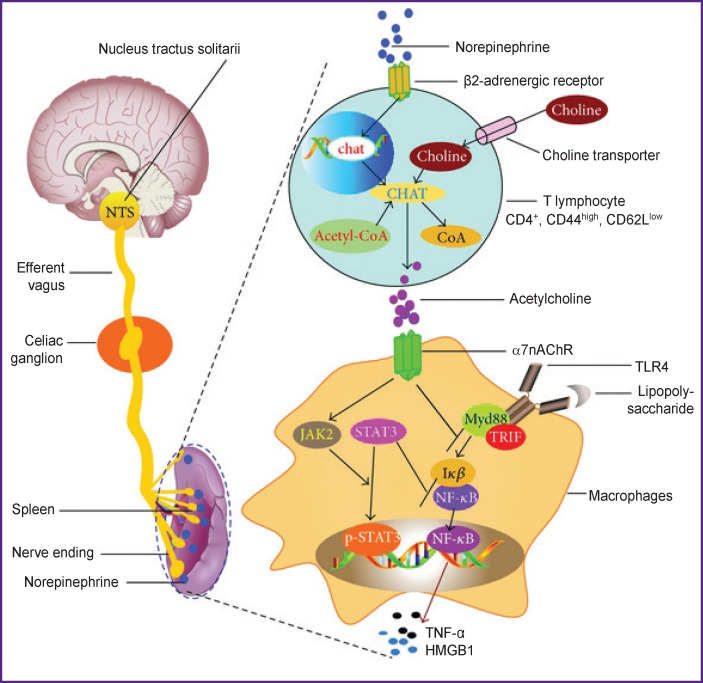
Scheme of intracellular transmission of anti-inflammatory sympathetic and parasympathetic signals in lymphocytes and macrophages [24]

Sympathetic and parasympathetic anti-inflammatory actions involve at least three intracellular signaling pathways. Regulation of intracellular signaling pathways in lymphocytes is critical for cell homeostasis and immune response. It has been shown that β2AR inhibits the production of NF-κB (nuclear factor κB), the transcription factor required for the expression of genes responsible for the synthesis of TNF-α and IL-1, thereby reducing the inflammatory response (the first intracellular mechanism) [21]. The second mechanism is represented by the intracellular signaling system JAK/STAT (Janus kinases/signal transducer and activator of transcription), which controls the subsequent cytokine production — the active elements of any inflammatory response. The JAK/STAT system incorporates Janus kinase, a signal transducer protein, and a transcription activator. This pathway transmits information from extracellular polypeptide signals through transmembrane receptors directly to the promoters of target genes in the nucleus, where they bind to the regulatory gene sequences and initiate their transcription [22]. Finally, the third intracellular signaling pathway is PI3K/AKT/mTOR that contains phosphoinositide-3-kinase enzymes (PI3K), alpha serine/threonine-protein kinases (AKT, also named protein kinase B), and mammalian target of rapamycin (mTOR). Evidence is provided for the association between IL-2 receptor and β2AR. Treatment of human lymphoid cell lines with the β2AR agonist isoproterenol alone (ISO) increases cAMP levels and mediates a stimulating response by activating AKT and extracellular-regulated kinase, which increases cell viability. Through this molecular mechanism, β2AR signaling can both stimulate and suppress lymphocyte responses, which may underlie the different immune responses to different therapeutic agents [23, 24] (Figure 1).

### Relationship between the ANS and intestinal microbiota.

**Figure 2:**
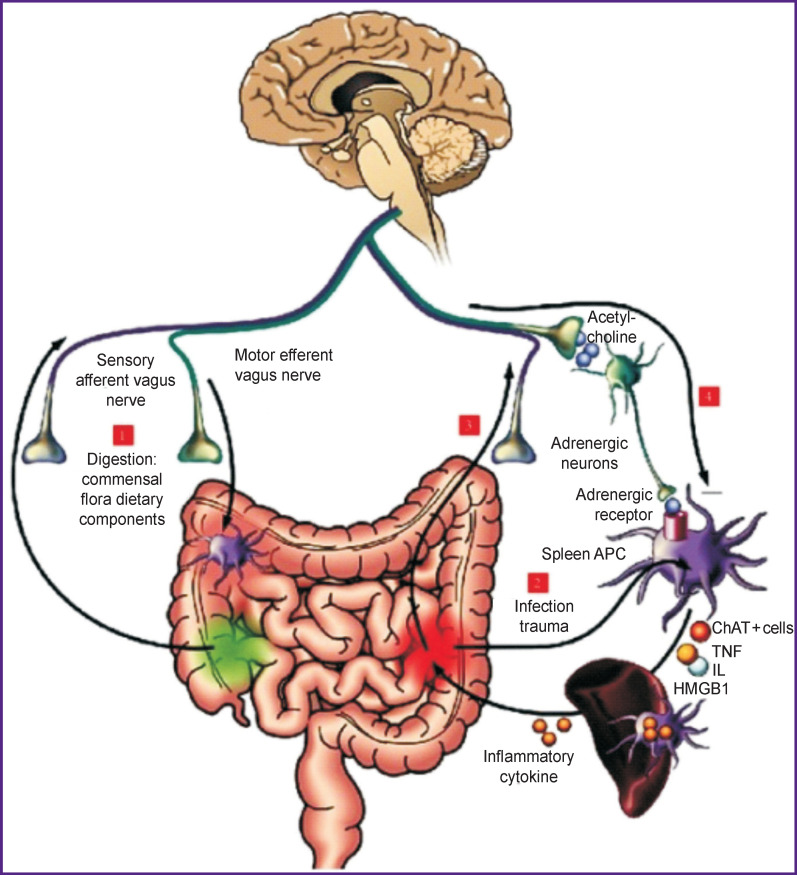
The interaction of the autonomic nervous system and the immune system of the gastrointestinal tract [30]

Their interaction is extremely important in the development of septic conditions. Vaughn et al. [25] showed that an energy-enriched diet modified the intestinal microbiota and, through the parasympathetic afferent transmission, disrupted the interaction between the brain and the intestines, leading to the accumulation of fat. About 100,000 billion bacteria populate the human intestines. The composition of this bacterial population depends on host age, body weight, and diet. Normally, the microbiota protects the body from pathogenic microorganisms and maintains the integral function of the intestinal wall, insulin sensitivity, metabolism, and, as has been found recently, mediates the interaction of the intestine with brain structures [26]. Lipopolysaccharides of gram-negative bacteria can penetrate the intestinal wall and reach the bloodstream. This process induces endotoxemia and inflammation and disrupts glucose metabolism thus resulting in insulin resistance, obesity, metabolic syndrome, type 2 diabetes, inflammation of the intestinal wall, autoimmune processes, and carcinogenesis. Norepinephrine, released from the terminal synapses of the ANS, can interfere with the protein synthesis in the cecum. Stress activates the hypothalamic-pituitary-adrenal (HPA) axis and the ANS increases the content of cortisol and pro-inflammatory cytokines, such as TNF-α, IL-8, IL-1β, and IL-6 [27–29]. An increase in the bacteria presence in the intestine and an excessive release of cytokines can disrupt the interaction between the HPA system, ANS and the intestine [30] (Figure 2).

### Heart rate variability analysis is the gold standard for assessing the functional state of the ANS.

Recent analyses of the sympathovagal involvement in sepsis emphasize the role of ANS as assessed by the heart rate variability (HRV). A meta-analysis of 2283 observations showed a negative correlation between the temporal parameters of HRV (SDNN), the high-frequency spectrum of HRV, and inflammatory markers [31]. Evidence was provided that an increase in the parasympathetic component (higher HRV) decreased inflammation through the CAP [32–34].

It is important to keep in mind one more aspect of systemic inflammation and the emergence of SIRS and MODS. The IL-1β, IL-6, and TNF-α are important in the development of the compensatory anti-inflammatory response syndrome (CARS), which prevents the development of SIRS [35]. The occurrence and progression of sepsis are caused by an inadequate response to infection that may lead to organ dysfunction and mortality. During sepsis, tissue damage leads to controlled complement activation, coagulation disorders, platelet dysfunction, and overproduction of cytokines. The balance between SIRS and CARS determines the outcome of sepsis [36]. Maintaining a balanced ANS response under these conditions is the basis for the adequate compensatory anti-inflammatory syndrome [37, 38]. It is important to control (including the digital navigation approach to HRV analysis) the ANS functions for the treatment and prevention of MODS and SIRS.

## Organ dysfunction and pathology of the ANS in sepsis

### The ANS and brain pathology

The main centers of the ANS are located in the brain: the hypothalamus, hippocampus, nucleus tractus solitarii, and the pituitary. Those structures are first to be damaged under various pathological conditions developing in this anatomical region (traumatic brain injury, cerebrovascular accident, anoxia, etc.). The brain is exposed to mediators of the systemic inflammatory response and reactive oxygen species. To date, autoimmune epilepsy, autoimmune anti-NMDA receptor encephalitis, autoimmune antibodies to cholinergic receptors of the somatic nervous system and the ANS have been described [39–41]. At the same time, microglia cells function as macrophages of the bone marrow hematopoiesis, and astroglia cells are able to synthesize active forms of oxygen and TNF-α. The microglia causes a cascade of reactions leading to secondary damage to the central nervous system. Gaddam et al. [42] noted that brain damage and dysfunction after traumatic brain injury caused systemic infection and moderate or severe organ damages in other anatomical areas. Frasch et al. [43] experimentally evaluated a correlation between HRV parameters and proinflammatory cytokine levels upon activation of the embryonic brain microglia under hypoxia. They found that the root mean square successive difference (RMSSD) between the duration of adjacent R–R intervals correlated with the level of IL-1β in the plasma (r=0.57; p=0.02; n=7), with HMGB1 (high mobility group box 1 protein) of the thalamic microglia (r=–0.94; p=0.005) and with the level of microglial cholinergic receptors of macrophages α7nAChR in the white matter of the brain (r=0.83; p=0.04). These results indicate the possibility of assessing the level of fetal neuroinflammation by measuring the HRV and also the emerging opportunities for non-invasive monitoring and targeted treatment. Of great interest are the results of Nicholls et al. [44] on the influence of norepinephrine on the functional activity of neutrophils via the adrenergic receptors. Neutrophils were isolated from the bone marrow of mice that received norepinephrine in different concentrations. Stroke was simulated and the neutrophil activity (cytokine-induced migration) was evaluated within 4 and 24 h. Treatment with norepinephrine for 4 h significantly reduced the neutrophil chemotaxis and also suppressed the production of interferon (IFN-γ) and IL-10; in addition, the neutrophil activity and phagocytosis decreased. These data show the importance of assessing the functional state of ANS, where norepinephrine can act as a blocker or inducer of systemic inflammation [45].

***Inflammatory bowel disease*** adversely affects the quality of life of millions worldwide. Although the etiology of the disease remains unclear, the aberrant activation of the immune system is considered the main cause. The most promising treatments are based on the selective inhibition of immune cells without causing excessive immunosuppression. One such treatment includes the inhibition of immune cell activation, which prevents the production of pro-inflammatory cytokines through neural stimulation. New therapeutic approaches are based on the discovery of the CAP — reflex arc, which induces the efferent transmission of vagal signals and reduces the activation of immune cells, which protects against mortality during sepsis and septic shock [46, 47]. Activation of CAP by stimulating the vagus has a protective effect on a wide variety of clinical disorders, including Crohn’s disease. The classical CAP pathway involves the activation of α7nAChR, positive macrophages of the spleen, using the positive β2AR CD4^+^ T cells. Stimulation by ultrasound or via the vagus activated the α7nAChR-positive peritoneal macrophages; consequently, the adaptive transfer of these activated macrophages reduced manifestations of colitis [48].

### Rheumatoid arthritis.

This is a complex chronic multisystem autoimmune disease that involves inflammation and the resulting vascular spasm, disturbance of osteoclastogenesis, and the ultimate destruction of bones and cartilage. One study [49] evaluated the expression and localization of the *α7nAChR* gene in major organs of rats with induced arthritis. Upon activation of the CAP pathway, mRNA expression decreased, which reduced the inflammation.

In ***chronic obstructive pulmonary disease (COPD),*** the cholinergic activity prevails, which (among other factors) restricts the air flow by contracting the smooth muscles of the respiratory tract. Therefore, blocking the contractile actions with anticholinergics is a useful therapeutic approach to reducing airflow restriction. In addition to the known effects of bronchoconstriction and mucus secretion, recent data obtained from animal models of COPD, suggest that acetylcholine plays a role in the COPD-associated inflammation. Since recently, combinations of long-acting β2-adrenergic agonists (LABAs) and long-acting muscarinic antagonists (LAMAs) have become available for the treatment of COPD. These double bronchodilators may have a synergistic anti-inflammatory effect, since β2AR stimulation induces inhibitory effects on inflammatory cells [50]. In addition to relaxing the airways, β2AR agonists may have additional anti-inflammatory effects [51].

***Bronchial asthma*** is characterized by an increasing number of inflammatory cells, especially eosinophils; in addition, the release of cytokines associated with T-helpers stimulates the production of reactive oxygen species. The CAP inhibits cytokine production and controls inflammation. A model of allergic asthma was used to study the neostigmine-induced activation of CAP and its effect on oxidative stress and airway inflammation. Activation of CAP with neostigmine reduced the levels of pro-inflammatory cytokines (IL-4, IL-5, IL-13, IL-1β, and TNF-α), which, in turn, reduced the inflow of eosinophils into the bronchial mucosa [52].

In ***kidneys and liver inflammations,*** CAP plays a therapeutic role as well. It is important to note that immune cells in the spleen express most of the components of the cholinergic system, such as acetylcholine, choline acetyltransferase, acetylcholinesterase, as well as muscarinic and nicotinic acetylcholine receptors; all the above provide for interaction between the systems. In general, this communication is able to suppress inflammation by using various mechanisms depending on cells involved [53, 54].

In ***obstetric disorders*** (e.g., preeclampsia), the CAP controls systemic inflammation by activating the α7nAChR, which are expressed in peripheral blood monocytes and macrophages [55]. Peripheral blood monocytes from 30 non-pregnant women (NP), 32 normotensive pregnant women (NT), and 35 women with preeclampsia (PE) were compared. The expression levels of α7nAChR protein and mRNA in monocytes from women with PE were significantly lower than those from the NP and NT groups (in both cases, p<0.01). The expression levels of α7nAChR protein in monocytes from the PE women negatively correlated with systolic blood pressure (r=–0.40; p=0.04), proteinuria (r=–0.54; p<0.01), TNF-α (r=–0.42; p=0.01), and IL-1β (r=–0.56; p<0.01), but positively correlated with IL-10 levels (r=0.43; p=0.01). The increase in the levels of TNF-α, IL-1β, and IL-6 was higher in the PE group than that in the NP and NT groups (all p<0.01), but the level of IL-10 in the PE group was lower than that in the NP and NT (p<0.01). In addition, the activity of NF-κB in monocytes from women with PE was higher than that in the NP and NT groups (p<0.01). These results suggest that suppression of α7nAChR may be associated with the development of preeclampsia by increasing the pro-inflammatory and decreasing the anti-inflammatory cytokines presence via the NF-κB pathway.

***Type 1 diabetes*** is an autoimmune disease caused by T cells; it is associated with the death of β cells of the pancreas and, consequently, with the loss of insulin production. In an experimental autoimmune disease, pretreatment with the specific acetylcholinesterase inhibitor (AChEI) paraoxon prevented the development of hyperglycemia in mice [56]. This effect correlated with the inhibition of T cell infiltration into the pancreatic islets and with the decrease in pro-inflammatory cytokines. Therefore, cholinergic stimulation may have a therapeutic effect on autoimmune diabetes.

***Obesity*** is a chronic condition associated with dysfunction of the ANS and HPA axis and mild inflammatory manifestations. Monocytes in animals with obesity caused by a high-fat diet had all pro-inflammatory cytokines at high expression levels and also a higher percentage of monocytes of a pro-inflammatory phenotype than that under a low-calorie diet. Moreover, β2-adrenergic stimulation in monocytes was anti-inflammatory only in obese animals in which the pro-inflammatory state was noted at the baseline [57, 58].

## Therapeutic interventions in ANS dysfunction and generalized infection

The cholinergic anti-inflammatory pathway is controlled through the vagus and prevents damage to cells and tissues caused by overproduction of cytokines. When the parasympathetic nervous system (vagal activity) is compromised, an inhibition of the CAP can develop. Normalization of the parasympathetic nervous system seems to be a new promising therapeutic approach aiming to suppress the systemic inflammatory changes and thereby improve the prognosis in patients with MODS and SIRS [59]. Medications able to eliminate the imbalance in the ANS are recognized as a new direction of intensive care [60, 61]. Changes in the production of pro-inflammatory cytokines in the presence of cholinesterase blockers (galantamine) or after vagotomy ultimately reduce the severity of endotoxemia and mortality during sepsis [62].

The pharmacological use of galantamine for cholinergic stimulation has been proved effective in attenuating the obesity-associated inflammation, neuroinflammation, and metabolic disorders [63]. Njoku et al. [64] showed that galantamine — an inhibitor of cholinesterase and a positive allosteric modulator of nAChRs — reduced the cognitive deficit after traumatic brain injury.

The CAP activation was also documented in the presence of another cholinesterase blocker, physostigmine [65]. The authors studied 20 patients with perioperative septic shock caused by intra-abdominal infection. The physostigmine group received an initial dose of 0.04 mg/kg physostigmine salicylate followed by a continuous infusion of 1 mg/h for 120 h (5 days); the placebo group was treated with 0.9% sodium chloride. The mean organ failure score (SOFA) was assessed during the treatment and 14 days after treatment. The SOFA values were 8.9±2.5 and 11.3±3.6 (mean ± SD) for the physostigmine and placebo groups, respectively. Given the age of the patients, the difference of the means was not statistically significant (–2.37; 95% CI: –5.43 to 0.70; p=0.121). The required doses of norepinephrine in the physostigmine group were lower (p=0.064); the faster decrease in heart rate indicated less hemodynamic instability.

The role of dexmedetomidine, an α2-adrenergic agonist causing either sympathetic inhibition or activation of CAP, has been documented. Dexmedetomidine prevents apoptosis of neurons and inhibits inflammation. The use of dexmedetomidine reduces the levels of protein S100β, neuron-specific enolase, and IL-6 in blood plasma and brain of experimental animals [66]. The expression of α7nAChR and IL-1β, TNF-α, S100β protein, and the brain-derived neurotrophic factor (BDNF) in animals treated with dexmedetomidine were studied by immunohistochemistry [67]. This α2-adrenergic agonist reduced the expression of α7nAChR, IL-1β, TNF-α, and S100β, and also increased the level of BDNF in the hippocampus. Systemic inflammation in rats, caused by an intraperitoneal injection of 5.0 mg/kg lipopolysaccharide, led to a loss of consciousness and the development of neuroinflammation in the hippocampus. In this case, dexmedetomidine prevented the inflammation-induced activation of microglia [68]. This α2-adrenergic agonist regulates gene expression, activation of cell channels, release of transmitters, inflammation, and cellular apoptosis [69–75].

Recent studies [76, 77] have shown that sepsis-induced cardiac dysfunction is caused (in a number of ways) by sympathetic nerve overstimulation. In a single-center randomized trial, it was shown that esmolol (selective β1-blocker) reduced mortality in patients who developed septic shock within 1 month after surgery. Blockage of β-receptors in sepsis reduces the production of pro-inflammatory cytokines, suppresses hypermetabolic status, maintains glucose balance, and reduces the manifestations of coagulopathy. A number of studies emphasized the crucial role of hypothalamic structures in controlling the peripheral immune system and inflammation [78–84].

Acetylcholine is a key anti-inflammatory transmitter of the cholinergic anti-inflammatory route. The relationship between the acetylcholine concentration in the blood plasma and the manifestations of inflammatory reactions was studied [85]. In a paper by Tao et al. [86], 113 patients were included in a prospective study. All of them completed the protocol of early enteral nutrition 24–48 h after admission to the intensive care unit. At the baseline and days 1, 3, 5, and 7, the levels of acetylcholine and the inflammation markers (TNF-α, IL-1β, and IL-6) were studied. Compared with the baseline (15.6±2.8 nmol/L), the plasma acetylcholine level significantly increased on day 3 — up to 18.6±6.7 nmol/L, on day 5 — up to 19.3±6.2 nmol/L, and on day 7 — up to 19.7±4.3 nmol/L (p<0.001). Compared to the baseline (176.2±50.4 pg/ml), the plasma TNF-α level significantly decreased on day 3 — to 144.0±77.4 pg/ml, on day 5 — 127.3±51.8 pg/ml, and on day 7 — up to 111.4±42.5 pg/ml (p<0.05). Compared with the baseline, the level of IL-1 in the plasma decreased significantly by day 7 (p<0.05), and that of IL-6 — on days 5 and 7 (p<0.05). The 28-day mortality rate was 28.3% (32/113). The elevated plasma acetylcholine levels correlated with a favorable prognosis in this critical condition.

Sympathomimetic transmitters can also simulate inflammatory reactions. Norepinephrine and adrenaline have been shown to dose-dependently suppress the release of IL-27 from activated macrophages and improve survival in septic shock [87]. The above data confirm the important role of ANS in acute inflammation. Lymphoid organs are enriched with sympathetic innervation where the secretion of noradrenaline (the β2-specific agonist) occurs. Immune cells contain adrenergic receptors, allowing the sympathetic nervous system to directly control the immune function. Norepinephrine can inhibit the production of the pro-inflammatory cytokine TNF-α and increase the production of the anti-inflammatory cytokine IL-10 by immune cells in response to lipopolysaccharide-induced endotoxemia. Thus, norepinephrine modifies systemic inflammation in sepsis [88]. The sympathomimetic phenylephrine can inhibit sepsis-induced cardiac dysfunction, inflammation, and mitochondrial damage by activating the intracellular PI3K/AKT/mTOR signaling pathway. In experimental peritonitis and sepsis caused by ligation and puncture of the cecum, phenylephrine reduced the production of TNF-α and IL-6 and also increased survival [89]. These and other data demonstrate that the manifestations of systemic inflammation are most pronounced with cholinergic or adrenergic dysfunction.

The results obtained to date indicate novel approaches to targeted therapy of ANS dysfunction in sepsis and other conditions of immune dysregulation [90–93]. It is crucial to determine the type of ANS dysfunction — in the form of sympathetic or parasympathetic hyperactivity — for subsequent targeted therapy of systemic inflammation using sympathomimetic or sympatholytic agents [94–98].

## Conclusion

**Figure 3:**
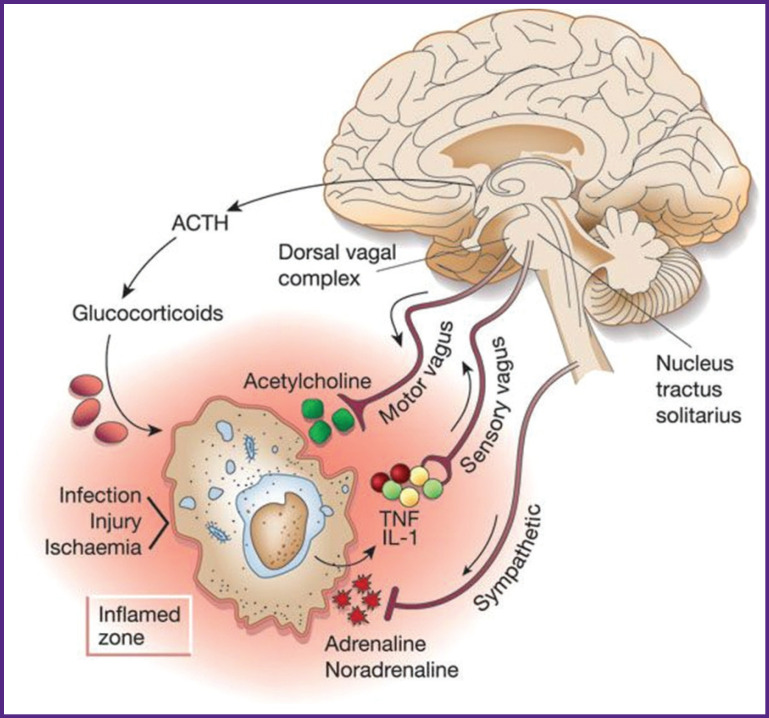
The anti-inflammatory mechanism developing in the autonomic nervous system during infection, damage, and ischemia [105]

Dysfunction of the autonomic nervous system in the brain during sepsis largely determines lethality and severity of systemic inflammation [99–104]. However, there are still no specific approaches to assessing the ANS dysfunction and developing specific therapies for its correction. This review clearly demonstrates that the ANS has an impact on the occurrence, course, and outcome of a generalized infection. The parasympathetic arm of the ANS is able to simulate inflammation through cholinergic receptors of internal organs, macrophages, and lymphocytes (the cholinergic anti-inflammatory pathway). The sympathetic part of the ANS also modifies the activity of macrophages and lymphocytes via β2-adrenergic receptors, stimulating the synthesis of β2AR-IL-10 cytokines. Thus, the interaction of the ANS, infectious agents, and the immune system ensures the maintenance of homeostasis or the appearance of a critical generalized infection [105] (Figure 3).

During inflammation, activation of the ANS simulates the inflammatory response through the release of sympathetic or parasympathetic neurotransmitters and neuropeptides. It is highly important to determine the functional state of the ANS in such critical conditions, since both cholinergic and sympathomimetic agents can act as either anti-inflammatory or pro-inflammatory stimuli. Maintaining a balanced response of the ANS under these conditions is the basis for the formation of CARS, which prevents the development of SIRS. By modifying the activity of the sympathetic and parasympathetic nervous system through blocking or activating the adrenergic or cholinergic receptors, it is possible to treat inflammation in sepsis. Today, it is important to determine the type of ANS dysfunction and apply the means of intensive care to treat it. Obviously, the activation of the cholinergic anti-inflammatory pathway and the inhibition of the cytokine production (as well as protecting against inflammatory damage during endotoxemia and acute sepsis) is most appropriate in the case of excessive sympathetic activity. In turn, the use of β2AR agonists is most appropriate to counteract the parasympathetic hyperactivity. Research into dysfunctions of the sympathetic and parasympathetic nervous system during inflammation is important to prospectively assess the intensive treatment strategies.

## References

[1] Williams D.P., Koenig J., Carnevali L., Sgoifo A., Jarczok M.N., Sternberg E.M., Thayer J.F (2019). Heart rate variability and inflammation: a meta-analysis of human studies.. Brain Behav Immun.

[2] Werdan K., Schmidt H., Ebelt H., Zorn-Pauly K., Koidl B., Hoke R.S., Heinroth K., Müller-Werdan U. (2009). Impaired regulation of cardiac function in sepsis, SIRS, and MODS.. Can J Physiol Pharmacol.

[3] Raithel D.S., Ohler K.H., Porto I., Bicknese A.R., Kraus D.M (2015). Morphine: an effective abortive therapy for pediatric paroxysmal sympathetic hyperactivity after hypoxic brain injury.. J Pediatr Pharmacol Ther.

[4] Baguley I.J., Perkes I.E., Fernandez-Ortega J.F., Rabinstein A.A., Dolce G., Hendricks H.T (2014). Consensus Working Group. Paroxysmal sympathetic hyperactivity after acquired brain injury: consensus on conceptual definition, nomenclature, and diagnostic criteria.. J Neurotrauma.

[5] Esterov D., Greenwald B.D. (2017). Autonomic dysfunction after mild traumatic brain injury.. Brain Sci.

[6] Wang D.W., Yin Y.M., Yao Y.M (2016). Vagal modulation of the inflammatory response in sepsis.. Int Rev Immunol.

[7] Zila I., Mokra D., Kopincova J., Kolomaznik M., Javorka M., Calkovska A (2017). Vagal-immune interactions involved in cholinergic anti-inflammatory pathway.. Physiol Res.

[8] Huang Y., Zhao C., Su X (2019). Neuroimmune regulation of lung infection and inflammation.. QJM.

[9] Ren C., Li X.H., Wang S.B., Wang L.X., Dong N., Wu Y., Yao Y.M (2018). Activation of central alpha 7 nicotinic acetylcholine receptor reverses suppressed immune function of T lymphocytes and protects against sepsis lethality.. Int J Biol Sci.

[10] Reyes-Lagos J.J., Ledesma-Ramírez C.I., Pliego-Carrillo A.C., Peña-Castillo M.Á., Echeverría J.C., Becerril-Villanueva E., Pavón L., Pacheco-López G. (2018). Neuroautonomic activity evidences parturition as a complex and integrated neuro-immune-endocrine process.. Ann N Y Acad Sci.

[11] Qian Y.S., Zhao Q.Y., Zhang S.J., Zhang Y.J., Wang Y.C., Zhao H.Y., Dai Z.X., Tang Y.H., Wang X., Wang T., Huang C.X (2018). Effect of α7nAChR mediated cholinergic anti-inflammatory pathway on inhibition of atrial fibrillation by low-level vagus nerve stimulation.. Zhonghua Yi Xue Za Zhi.

[12] Murray K., Reardon C (2018). The cholinergic anti-inflammatory pathway revisited.. Neurogastroenterol Motil.

[13] Yamada M., Ichinose M (2018). The cholinergic anti-inflammatory pathway: an innovative treatment strategy for respiratory diseases and their comorbidities.. Curr Opin Pharmacol.

[14] Pavlov V.A., Ochani M., Yang L.H., Gallowitsch-Puerta M., Ochani K., Lin X., Levi J., Parrish W.R., Rosas-Ballina M., Czura C.J., Larosa G.J., Miller E.J., Tracey K.J., Al-Abed Y (2007). Selective α7-nicotinic acetylcholine receptor agonist GTS-21 improves survival in murine endotoxemia and severe sepsis.. Crit Care Med.

[15] Lu J., Goh S.J., Tng P.Y., Deng Y.Y., Ling E.A., Moochhala S (2009). Systemic inflammatory response following acute traumatic brain injury.. Front Biosci (Landmark Ed).

[16] Shin S.S., Dixon C.E (2015). Alterations in cholinergic pathways and therapeutic strategies targeting cholinergic system after traumatic brain injury.. J Neurotrauma.

[17] Liu Q., Xie J., Yang Y (2019). Advances in the regulation mechanism of cholinergic anti-inflammatory pathway on sepsis.. Zhonghua Wei Zhong Bing Ji Jiu Yi Xue.

[18] Guyot M., Simon T., Panzolini C., Ceppo F., Daoudlarian D., Murris E., Macia E., Abélanet S., Sridhar A., Vervoordeldonk M.J., Glaichenhaus N., Blancou P (2019). Apical splenic nerve electrical stimulation discloses an anti-inflammatory pathway relying on adrenergic and nicotinic receptors in myeloid cells.. Brain Behav Immun.

[19] Noh H., Yu M.R., Kim H.J., Lee J.H., Park B.W., Wu I., Matsumoto M., King G.L (2017). Beta 2 adrenergic receptor agonists are novel regulators of macrophage activation in diabetic renal and cardiovascular complications.. Kidney Int.

[20] Lechtenberg K.J., Meyer S.T., Doyle J.B., Peterson T.C., Buckwalter M.S (2019). Augmented β2-adrenergic signaling dampens the neuroinflammatory response following ischemic stroke and increases stroke size.. J Neuroinflammation.

[21] Zabrodskii P.F., Gromov M.S., Maslyakov V.V (2018). Combined effect of NF-κB inhibitor and β2-adrenoreceptor agonist on mouse mortality and blood concentration of proinflammatory cytokines in sepsis.. Bull Exp Biol Med.

[22] Sun J.J., Lan J.F., Zhao X.F., Vasta G.R., Wang J.X (2017). Binding of a C-type lectin’s coiled-coil domain to the Domeless receptor directly activates the JAK/STAT pathway in the shrimp immune response to bacterial infection.. PLoS Pathog.

[23] Ruiz-Medina B.E., Cadena-Medina D.A., Esparza E., Arrieta A.J., Kirken R.A. (2018). Isoproterenol-induced beta-2 adrenergic receptor activation negatively regulates interleukin-2 signaling.. Biochem J.

[24] Wu H., Li L., Su X (2014). Vagus nerve through α7 nAChR modulates lung infection and inflammation: models, cells, and signals.. Biomed Res Int.

[25] Vaughn A.C., Cooper E.M., DiLorenzo P.M., O’Loughlin L.J., Konkel M.E., Peters J.H., Hajnal A., Sen T., Lee S.H., de La Serre C.B., Czaja K. (2017). Energy-dense diet triggers changes in gut microbiota, reorganization of gut brain vagal communication and increases body fat accumulation.. Acta Neurobiol Exp (Wars).

[26] Cawthon C.R., de La Serre C.B. (2018). Gut bacteria interaction with vagal afferents.. Brain Res.

[27] Bonaz B., Sinniger V., Pellissier S (2019). Vagus nerve stimulation at the interface of brain-gut interactions.. Cold Spring Harb Perspect Med.

[28] Houlden A., Goldrick M., Brough D., Vizi E.S., Lenart N., Martinecz B., Roberts I.S., Denes A (2016). Brain injury induces specific changes in the caecal microbiota of mice via altered autonomic activity and mucoprotein production.. Brain Behav Immun.

[29] Kigerl K.A., Mostacada K., Popovich P.G. (2018). Gut microbiota are disease-modifying factors after traumatic spinal cord injury.. Neurotherapeutics.

[30] de Jonge W.J. (2013). The gut’s little brain in control of intestinal immunity.. ISRN Gastroenterol.

[31] Radmark L., Sidorchuk A., Osika W., Niemi M (2019). A systematic review and meta-analysis of the impact of mindfulness based interventions on heart rate variability and inflammatory markers.. J Clin Med.

[32] Sharma V.K., Savitha S., Vinod K.V., Rajappa M., Subramanian S.K., Rajendran R (2019). Assessment of autonomic functions and its association with telomerase level, oxidative stress and inflammation in complete glycemic spectrum — an exploratory study.. Diabetes Metab Syndr.

[33] Pong J.Z., Fook-Chong S., Koh Z.X., Samsudin M.I., Tagami T., Chiew C.J., Wong T.H., Ho A.F.W., Ong M.E.H., Liu N (2019). Combining heart rate variability with disease severity score variables for mortality risk stratification in septic patients presenting at the emergency department.. Int J Environ Res Public Health.

[34] Rupprecht S., Finn S., Hoyer D., Guenther A., Witte O.W., Schultze T., Schwab M. (2020). Association between systemic inflammation, carotid arteriosclerosis, and autonomic dysfunction.. Transl Stroke Res.

[35] Magrone T., Jirillo E (2019). Sepsis: from historical aspects to novel vistas. pathogenic and therapeutic considerations.. Endocr Metab Immune Disord Drug Targets.

[36] Kanashiro A., Sônego F., Ferreira R.G., Castanheira F.V., Leite C.A., Borges V.F., Nascimento D.C., Cólon D.F., Alves-Filho J.C., Ulloa L., Cunha F.Q (2017). Therapeutic potential and limitations of cholinergic anti-inflammatory pathway in sepsis.. Pharmacol Res.

[37] Assinger A., Schrottmaier W.C., Salzmann M., Rayes J (2019). Platelets in sepsis: an update on experimental models and clinical data.. Front Immunol.

[38] Vergadi E., Vaporidi K., Tsatsanis C (2018). Regulation of endotoxin tolerance and compensatory anti-inflammatory response syndrome by non-coding RNAs.. Front Immunol.

[39] Quek A.M.L., Britton J.W., McKeon A., So E., Lennon V.A., Shin C., Klein C., Watson R.E., Kotsenas A.L., Lagerlund T.D., Cascino G.D., Worrell G.A., Wirrell E.C., Nickels K.C., Aksamit A.J., Noe K.H., Pittock S.J (2012). Autoimmunne epilepsy: clinical characteristics and response to immunotherapy.. Arch Neurol.

[40] Bauer J., Becker A.J., Elyaman W., Peltola J., Rüegg S., Titulaer M.J., Varley J.A., Beghi E (2017). Innate and adaptive immunity in human epilepsies.. Epilepsia.

[41] Golden E.P., Vernino S (2019). Autoimmune autonomic neuropathies and ganglionopathies: epidemiology, pathophysiology, and therapeutic advances.. Clin Auton Res.

[42] Gaddam S.S., Buell T., Robertson C.S (2015). Systemic manifestations of traumatic brain injury.. Handb Clin Neurol.

[43] Frasch M.G., Szynkaruk M., Prout A.P., Nygard K., Cao M., Veldhuizen R., Hammond R., Richardson B.S (2016). Decreased neuroinflammation correlates to higher vagus nerve activity fluctuations in near-term ovine fetuses: a case for the afferent cholinergic anti-inflammatory pathway?. J Neuroinflammation.

[44] Nicholls A.J., Wen S.W., Hall P., Hickey M.J., Wong C.H.Y (2018). Activation of the sympathetic nervous system modulates neutrophil function.. J Leukoc Biol.

[45] Oikawa S., Kai Y., Mano A., Sugama S., Mizoguchi N., Tsuda M., Muramoto K., Kakinuma Y (2019). Potentiating a non-neuronal cardiac cholinergic system reinforces the functional integrity of the blood brain barrier associated with systemic anti-inflammatory responses.. Brain Behav Immun.

[46] Boeckxstaens G (2013). The clinical importance of the anti-inflammatory vagovagal reflex.. Handb Clin Neurol.

[47] Nunes N.S., Chandran P., Sundby M., Visioli F., da Costa Gonçalves F., Burks S.R., Paz A.H., Frank J.A. (2019). Therapeutic ultrasound attenuates DSS-induced colitis through the cholinergic anti-inflammatory pathway.. EBioMedicine.

[48] Inoue T., Abe C., Kohro T., Tanaka S., Huang L., Yao J., Zheng S., Ye H., Inagi R., Stornetta R.L., Rosin D.L., Nangaku M., Wada Y., Okusa M.D (2019). Non-canonical cholinergic anti-inflammatory pathway-mediated activation of peritoneal macrophages induces Hes1 and blocks ischemia/reperfusion injury in the kidney.. Kidney Int.

[49] Li Z., Hao H., Gao Y., Wang Z., Lu W., Liu J (2019). Expression and localization analyses of the cholinergic anti-inflammatory pathway and α7nAchR in different tissues of rats with rheumatoid arthritis.. Acta Histochem.

[50] Yamada M., Ichinose M (2018). The cholinergic pathways in inflammation: a potential pharmacotherapeutic target for COPD.. Front Pharmacol.

[51] Tian Y., Miao B., Charles E.J., Wu D., Kron I.L., French B.A., Yang Z (2018). Stimulation of the beta2 adrenergic receptor at reperfusion limits myocardial reperfusion injury via an interleukin-10-dependent anti-inflammatory pathway in the spleen.. Circ J.

[52] Antunes G.L., Silveira J.S., Kaiber D.B., Luft C., da Costa M.S., Marques E.P., Ferreira F.S., Breda R.V., Wyse A.T.S., Stein R.T., Pitrez P.M., da Cunha A.A. (2020). Cholinergic anti-inflammatory pathway confers airway protection against oxidative damage and attenuates inflammation in an allergic asthma model.. J Cell Physiol.

[53] Jarczyk J., Yard B.A., Hoeger S (2019). The cholinergic anti-inflammatory pathway as a conceptual framework to treat inflammation-mediated renal injury.. Kidney Blood Press Res.

[54] Hajiasgharzadeh K., Baradaran B (2017). Cholinergic anti-inflammatory pathway and the liver.. Adv Pharm Bull.

[55] Xu H., Shi Q., Mo Y., Wu L., Gu J., Xu Y (2019). Downregulation of α7 nicotinic acetylcholine receptors in peripheral blood monocytes is associated with enhanced inflammation in preeclampsia.. BMC Pregnancy Childbirth.

[56] Fernández-Cabezudo M.J., George J.A., Bashir G., Mohamed Y.A., Al-Mansori A., Qureshi M.M., Lorke D.E., Petroianu G., Al-Ramadi B.K. (2019). Involvement of acetylcholine receptors in cholinergic pathway-mediated protection against autoimmune diabetes.. Front Immunol.

[57] Gálvez I., Martín-Cordero L., Hinchado M.D. (2019). Álvarez-Barrientos A., Ortega E. Anti-inflammatory effect of β2 adrenergic stimulation on circulating monocytes with a pro-inflammatory state in high-fat diet-induced obesity.. Brain Behav Immun.

[58] Ortega E., Gálvez I., Martín-Cordero L (2019). Adrenergic regulation of macrophage-mediated innate/inflammatory responses in obesity and exercise in this condition: role of β2 adrenergic receptors.. Endocr Metab Immune Disord Drug Targets.

[59] Hatakeyama N., Matsuda N (2014). Alert cell strategy: mechanisms of inflammatory response and organ protection.. Curr Pharm Des.

[60] Clar D.T., Sharma S (2019). Autonomic pharmacology.. StatPearls..

[61] Samuel S., Allison T.A., Lee K., Choi H.A (2016). Pharmacologic management of paroxysmal sympathetic hyperactivity after brain injury.. J Neurosci Nurs.

[62] Lehner K.R., Silverman H.A., Addorisio M.E., Roy A., Al-Onaizi M.A., Levine Y., Olofsson P.S., Chavan S.S., Gros R., Nathanson N.M., Al-Abed Y., Metz C.N., Prado V.F., Prado M.A.M., Tracey K.J., Pavlov V.A. (2019). Forebrain cholinergic signaling regulates innate immune responses and inflammation.. Front Immunol.

[63] Chang E.H., Chavan S.S., Pavlov V.A (2019). Cholinergic control of inflammation, metabolic dysfunction, and cognitive impairment in obesity-associated disorders: mechanisms and novel therapeutic opportunities.. Front Neurosci.

[64] Njoku I., Radabaugh H.L., Nicholas M.A., Kutash L.A., O’Neil D.A., Marshall I.P., Cheng J.P., Kline A.E., Bondi C.O (2019). Chronic treatment with galantamine rescues reversal learning in an attentional set-shifting test after experimental brain trauma.. Exp Neurol.

[65] Pinder N., Bruckner T., Lehmann M., Motsch J., Brenner T., Larmann J., Knebel P., Hoppe-Tichy T., Swoboda S., Weigand M.A., Hofer S., Zimmermann J.B (2019). Effect of physostigmine on recovery from septic shock following intra-abdominal infection — results from a randomized, double-blind, placebo-controlled, monocentric pilot trial (Anticholium® per Se).. J Crit Care.

[66] Chen Y., Zhang X., Zhang B., He G., Zhou L., Xie Y (2017). Dexmedetomidine reduces the neuronal apoptosis related to cardiopulmonary bypass by inhibiting activation of the JAK2-STAT3 pathway.. Drug Des Devel Ther.

[67] Xu K.L., Liu X.Q., Yao Y.L., Ye M.R., Han Y.G., Zhang T., Chen G., Lei M (2018). Effect of dexmedetomidine on rats with convulsive status epilepticus and association with activation of cholinergic anti-inflammatory pathway.. Biochem Biophys Res Commun.

[68] Yamanaka D., Kawano T., Nishigaki A., Aoyama B., Tateiwa H., Shigematsu-Locatelli M., Locatelli F.M., Yokoyama M (2017). Preventive effects of dexmedetomidine on the development of cognitive dysfunction following systemic inflammation in aged rats.. J Anesth.

[69] Cai Y., Xu H., Yan J., Zhang L., Lu Y (2014). Molecular targets and mechanism of action of dexmedetomidine in treatment of ischemia/reperfusion injury.. Mol Med Rep.

[70] Zhang J., Xia F., Zhao H., Peng K., Liu H., Meng X., Chen C., Ji F (2019). Dexmedetomidine-induced cardioprotection is mediated by inhibition of high mobility group box-1 and the cholinergic anti-inflammatory pathway in myocardial ischemia-reperfusion injury.. PLoS One.

[71] Jiang L., Hu M., Lu Y., Cao Y., Chang Y., Dai Z (2017). The protective effects of dexmedetomidine on ischemic brain injury: a meta-analysis.. J Clin Anesth.

[72] Hu J., Vacas S., Feng X., Lutrin D., Uchida Y., Lai I.K., Maze M (2018). Dexmedetomidine prevents cognitive decline by enhancing resolution of high mobility group box 1 protein-induced inflammation through a vagomimetic action in mice.. Anesthesiology.

[73] Janssen T.L., Alberts A.R., Hooft L., Mattace-Raso F., Mosk C.A., van der Laan L. (2019). Prevention of postoperative delirium in elderly patients planned for elective surgery: systematic review and meta-analysis.. Clin Interv Aging.

[74] Lankadeva Y.R., Ma S., Iguchi N., Evans R.G., Hood S.G., Farmer D.G.S., Bailey S.R., Bellomo R., May C.N (2019). Dexmedetomidine reduces norepinephrine requirements and preserves renal oxygenation and function in ovine septic acute kidney injury.. Kidney Int.

[75] Zi S.F., Li J.H., Liu L., Deng C., Ao X., Chen D.D., Wu S.Z. (2019). Dexmedetomidine-mediated protection against septic liver injury depends on TLR4/MyD88/NF-κB signaling downregulation partly via cholinergic anti-inflammatory mechanisms.. Int Immunopharmacol.

[76] Suzuki T., Suzuki Y., Okuda J., Kurazumi T., Suhara T., Ueda T., Nagata H., Morisaki H (2017). Sepsis-induced cardiac dysfunction and β-adrenergic blockade therapy for sepsis.. J Intensive Care.

[77] Brown S.M., Beesley S.J., Lanspa M.J., Grissom C.K., Wilson E.L., Parikh S.M., Sarge T., Talmor D., Banner-Goodspeed V., Novack V., Thompson B.T., Shahul S (2018). Esmolol to Control Adrenergic Storm in Septic Shock-ROLLIN (ECASSS-R) study. Esmolol infusion in patients with septic shock and tachycardia: a prospective, single-arm, feasibility study.. Pilot Feasibility Study.

[78] Breit S., Kupferberg A., Rogler G., Hasler G (2018). Vagus nerve as modulator of the brain-gut axis in psychiatric and inflammatory disorders.. Front Psychiatry.

[79] Carod-Artal F.J. (2017). Clin Auton Res.

[80] Han C., Rice M.W., Cai D (2016). Neuroinflammatory and autonomic mechanisms in diabetes and hypertension.. Am J Physiol Endocrinol Metab.

[81] Gao H., Molinas A.J.R., Miyata K., Qiao X., Zsombok A (2017). Overactivity of liver-related neurons in the paraventricular nucleus of the hypothalamus: electrophysiological findings in db/db mice.. J Neurosci.

[82] Hong G.S., Zillekens A., Schneiker B., Pantelis D., de Jonge W.J., Schaefer N., Kalff J.C., Wehner S. (2019). Non-invasive transcutaneous auricular vagus nerve stimulation prevents postoperative ileus and endotoxemia in mice.. Neurogastroenterol Motil.

[83] Bosmans G., Appeltans I., Stakenborg N., Gomez-Pinilla P.J., Florens M.V., Aguilera-Lizarraga J., Matteoli G., Boeckxstaens G.E (2019). Vagus nerve stimulation dampens intestinal inflammation in a murine model of experimental food allergy.. Allergy.

[84] Papaioannou V., Pnevmatikos I (2019). Heart rate variability: a potential tool for monitoring immunomodulatory effects of parenteral fish oil feeding in patients with sepsis.. Nutr Metab Insights.

[85] Hoover В.B. (2017). Cholinergic modulation of the immune system presents new approaches for treating inflammation.. Pharmacol Ther.

[86] Tao G., Min-Hua C., Feng-Chan X., Yan C., Ting S., Wei-Qin L., Wen-Kui Y (2019). Changes of plasma acetylcholine and inflammatory markers in critically ill patients during early enteral nutrition: a prospective observational study.. J Crit Care.

[87] Roewe J., Higer M., Riehl D.R., Gericke A., Radsak M.P., Bosmann M (2017). Neuroendocrine modulation of IL-27 in macrophages.. J Immunol.

[88] Ağaç D., Estrada L.D., Maples R., Hooper L.V., Farrar J.D. (2018). The β2-adrenergic receptor controls inflammation by driving rapid IL-10 secretion.. Brain Behav Immun.

[89] Li H.M., Li K.Y., Xing Y., Tang X.X., Yang D.M., Dai X.M., Lu D.X., Wang H.D (2019). Phenylephrine attenuated sepsis-induced cardiac inflammation and mitochondrial injury through an effect on the PI3K/Akt signaling pathway.. J Cardiovasc Pharmacol.

[90] Mogilevski T., Burgell R., Aziz Q., Gibson P.R (2019). Review article: the role of the autonomic nervous system in the pathogenesis and therapy of IBD.. Aliment Pharmacol Ther.

[91] Sonnenberg G.F., Hepworth M.R. (2019). Functional interactions between innate lymphoid cells and adaptive immunity.. Nat Rev Immunol.

[92] Serhan C.N., de la Rosa X., Jouvene C. (2019). Novel mediators and mechanisms in the resolution of infectious inflammation: evidence for vagus regulation.. J Intern Med.

[93] Carnagarin R., Matthews V., Zaldivia M.T.K., Schlaich M.P (2019). The bidirectional interaction between the sympathetic nervous system and immune mechanisms in the pathogenesis of hypertension.. Br J Pharmacol.

[94] Reardon C (2016). Neuro-immune interactions in the cholinergic anti-inflammatory reflex.. Immunol Lett.

[95] Eduardo C.-R.C., Alejandra T.-I.G., Guadalupe D.-R.K.J., Herminia V.-R.G., Lenin P., Enrique B.-V., Evandro B.M., Oscar B., Iván G.-P.M (2019). Modulation of the extraneuronal cholinergic system on main innate response leukocytes.. J Neuroimmunol.

[96] Pereira M.R., Leite P.E (2016). The involvement of parasympathetic and sympathetic nerve in the inflammatory reflex.. J Cell Physiol.

[97] Kim H.G., Cheon E.J., Bai D.S., Lee Y.H., Koo B.H (2018). Stress and heart rate variability: a meta-analysis and review of the literature.. Psychiatry Investig.

[98] Liu L., Zhao M., Yu X., Zang W (2019). Pharmacological modulation of vagal nerve activity in cardiovascular diseases.. Neurosci Bull.

[99] Crippa I.A., Subirà C., Vincent J.L., Fernandez R.F., Hernandez S.C., Cavicchi F.Z., Creteur J., Taccone F.S. (2018). Impaired cerebral autoregulation is associated with brain dysfunction in patients with sepsis.. Crit Care.

[100] Sanz D., D’Arco F., Robles C.A., Brierley J (2018). Incidence and pattern of brain lesions in paediatric septic shock patients.. Br J Radiol.

[101] Esen F., Orhun G., Özcan P.E., Brenes Bastos A.R., Tüzün E. (2020). Diagnosing acute brain dysfunction due to sepsis.. Neurol Sci.

[102] Günther A., Schubert J., Witte O.W., Brämer D. (2019). Intensive care aspects of autoimmune encephalitis.. Med Klin Intensivmed Notfmed.

[103] Bonjorno Junior J.C., Caruso F.R., Mendes R.G., da Borghi-Silva A. (2019). Noninvasive measurements of hemodynamic, autonomic and endothelial function as predictors of mortality in sepsis: a prospective cohort study.. PLoS One.

[104] Biteker F.S., Özlek B., Çelik O., Özlek E., Çil C., Doğan V., Biteker M. (2018). Autonomic imbalance in sepsis.. Am J Emerg Med.

[105] Tracey K.J (2002). The inflammatory reflex.. Nature.

